# Does surgical treatment produce better outcomes than conservative treatment for acute primary patellar dislocations? A meta-analysis of 10 randomized controlled trials

**DOI:** 10.1186/s13018-020-01634-5

**Published:** 2020-03-24

**Authors:** Xuewu Xing, Hongyu Shi, Shiqing Feng

**Affiliations:** 1 Department of Orthopaedics, Tianjin First Central Hospital, First Central Clinical College of Tianjin Medical University, Tianjin, China; 2 Department of Orthopaedics, Tianjin Medical University General Hospital, Tianjin, China

**Keywords:** Patella, Dislocation, Primary, Acute, Surgery, Conservative, Treatment, MPFL

## Abstract

**Purpose:**

The objective of this study was to conduct the latest meta-analysis of randomized controlled trials (RCTs) that compare clinical results between surgery and conservative therapy of acute primary patellar dislocation (APPD), focusing on medial patellofemoral ligament (MPFL) reconstruction.

**Methods:**

We performed a literature search in Embase, The Cochrane Library, PubMed, and Medline to identify RCTs comparing APPD surgical treatment with conservative treatment from the establishment of each database to January 2019. The methodological quality of each RCT was assessed independently by the two authors through the Cochrane Collaboration’s “Risk of Bias” tool. Mean differences of continuous variables and risk ratios of dichotomous variables were computed for the pooled data analyses. The *I*^2^ statistic and the *χ*^2^ test were used to evaluate heterogeneity, with the significance level set at *I*^2^ > 50% or *P* < 0.10.

**Results:**

Ten RCTs with a sum of 569 patients (297 receiving surgical treatment and 263 receiving conservative treatment) met the inclusion criteria for meta-analysis. Pooled data analysis showed no statistical difference in the field of subluxation rate, Kujala score, patient satisfaction, and frequency of reoperation between the two groups. Tegner activity score and recurrent dislocation rate in the conservative group were significantly higher than those in the surgically treated group.

**Conclusions:**

Conservative treatment may produce better outcomes than surgery for APPD in consideration of Tegner activity score. However, in view of limited research available, the interpretation of the discoveries should be cautious. More convincing evidence is required to confirm the effect of MPFL reconstruction.

## Introduction

Acute primary patellar dislocation (APPD) accounts for 2–3% of knee injuries [1], and mostly occurs in adolescents and active people [2]. The incidence of APPD is 5.8–42 per 100,000 [3, 4]. Patellar dislocation can be caused by trauma or anatomical abnormalities [5, 6]. Lateral patellar dislocation is most common, resulting in rupture of the medial soft tissue [7], peculiarly the medial patellofemoral ligament (MPFL) [8], which leads to knee pain, patellar instability, and patellofemoral osteoarthritis [9]. MPFL provides 50–70% soft-tissue restraint in preventing patellar dislocation and is thus considered to be the primary soft tissue for patellar stabilization [10].

Conservative treatment, which consists of immobilization, physical therapy, and functional exercises, is traditionally the preferred option for APPD without an osteochondral fracture [11]. Nevertheless, it results in a recurrent dislocation rate of as high as 50% [1]. Therefore, surgical treatment is gradually gaining the attention of clinicians [8]. MPFL and its reconstruction have been considered as the greatest advance in the therapy of lateral patellar dislocation in the past decade [12], and achieve satisfactory outcomes such as a low recurrent dislocation rate [13]. Although reconstructive surgery has many advantages, it is also associated with patellar fracture, postoperative stiffness, medial knee pain, and failed reconstruction [14]. Therefore, it remains unclear which treatment is more advantageous.

Clinical randomized controlled trials (RCTs) have been conducted to compare surgery versus conservative treatment for APPD; the treatment that can best improve the clinical outcome is still debatable. Although, meta-analyses of related RCTs have been conducted [15–18], the latest one was published 3 years ago [19]. To offer the best evidence-based evidence available to the clinic, we performed the latest meta-analysis of randomized controlled trials (RCTs) that compare clinical results between surgery and conservative therapy of acute primary patellar dislocation (APPD), focusing on medial patellofemoral ligament (MPFL) reconstruction.

## Methods

### Study design and search strategy

This study was carried out in the light of the Preferred Reporting Items for Systematic Reviews and Meta-Analyses (PRISMA) guidelines (www.prisma-statement.org). We performed a literature search in Embase, The Cochrane Library, PubMed, and Medline to recognize relevant published articles from each database’s inception to January 2019. The following key words and related search terms were used: “patellar dislocation” “patella” “surgical procedure” “operative” “non-surgical” “non-operative,” and “conservative treatment.” Searching strategy was constructed by combining the above terms with “AND” or “OR.” There were no language restrictions. Additional manual searches were made for the list of references for all retrieved articles to identify potential-related studies.

### Study selection

All studies identified through literature searches were examined and chosen in accordance with the following inclusion criteria: (1) patients with APPD, (2) RCTs comparing a surgery group with a conservative treatment group, and (3) reporting of clinical outcomes of surgical or conservative management. There were no restrictions on the surgical techniques or conservative treatment strategies. Exclusion criteria included the following: (1) retrospective studies, cohort studies, and trials without a RCT design; (2) comparison of different operative procedures; and (3) experimental trials on animals or cadavers, single case reports, reviews, comments, editorials, guidelines, protocols, letters, and publications based on surgical registries.

### Data extraction

Data extraction was done by two reviewers independently. Any disputes were resolved through consultations. Data extracted from the included studies consisted of the first author, publication year, and follow-up duration, number, age, and gender of patients, surgical interventions, and evaluation indicators including the recurrent dislocation rate, subluxation rate, Kujala score, Tegner activity score, patient satisfaction, and frequency of reoperation. Data should be obtained whenever possible by contacting corresponding authors in the event of data loss.

### Assessment of study quality

The methodological quality of each RCT was assessed independently by the two authors through the Cochrane Collaboration’s “Risk of Bias” tool, including blinding (personnel, participants, and outcome assessors), randomization (allocation concealment and sequence generation), selection of outcomes reported, and completeness of outcome data. According to the above assessment, the risk of bias was categorized as low, unclear, or high for each of the included RCTs.

### Statistical analysis

Review Manager 5.3 was adopted for statistical analysis. The *χ*^2^ test and the *I*^2^ statistic were used to assess heterogeneity, with the significance level set at *P* < 0.10 or *I*^2^ > 50%. Publication bias was evaluated by funnel plot. If there was no heterogeneity, the fixed effect model was used. On the contrary, if heterogeneity existed, random effect model was adopted. The mean difference (MD) and the 95% confidence intervals (CI) were calculated for continuous variables. Dichotomous outcomes were expressed as a risk ratio (RR) with 95% CIs. The statistical difference was considered significant if *P* < 0.05. Outcomes were expressed using forest plots.

## Results

### Search outcomes

**Fig. 1:**
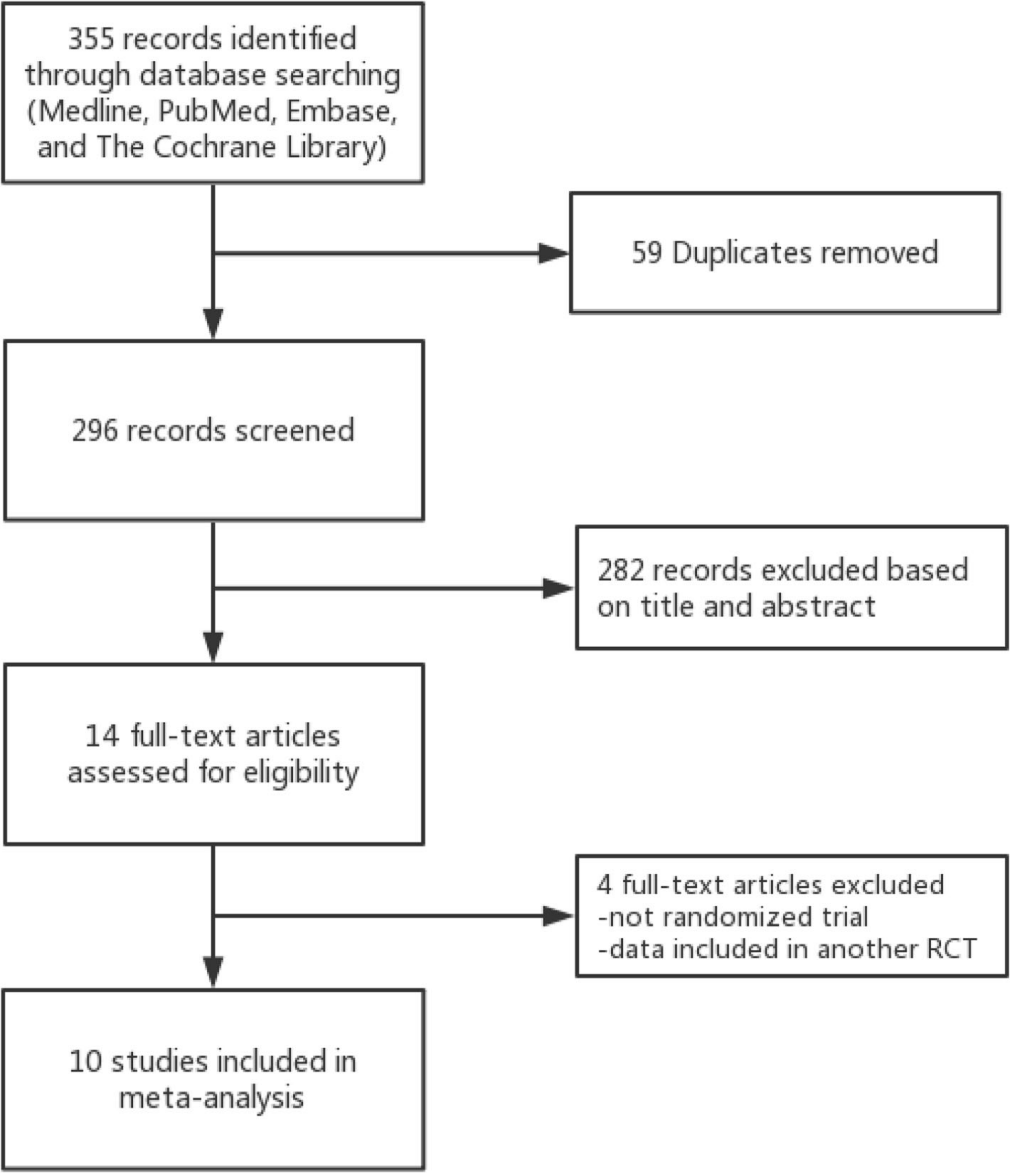
PRISMA flowchart of eligible studies selection

**Fig. 2:**
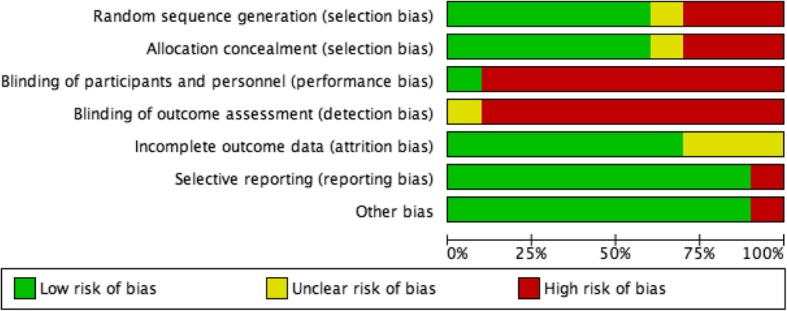
Risk of bias graph: review authors’ judgements about each risk of bias item presented as percentages across all included studies

**Fig. 3:**
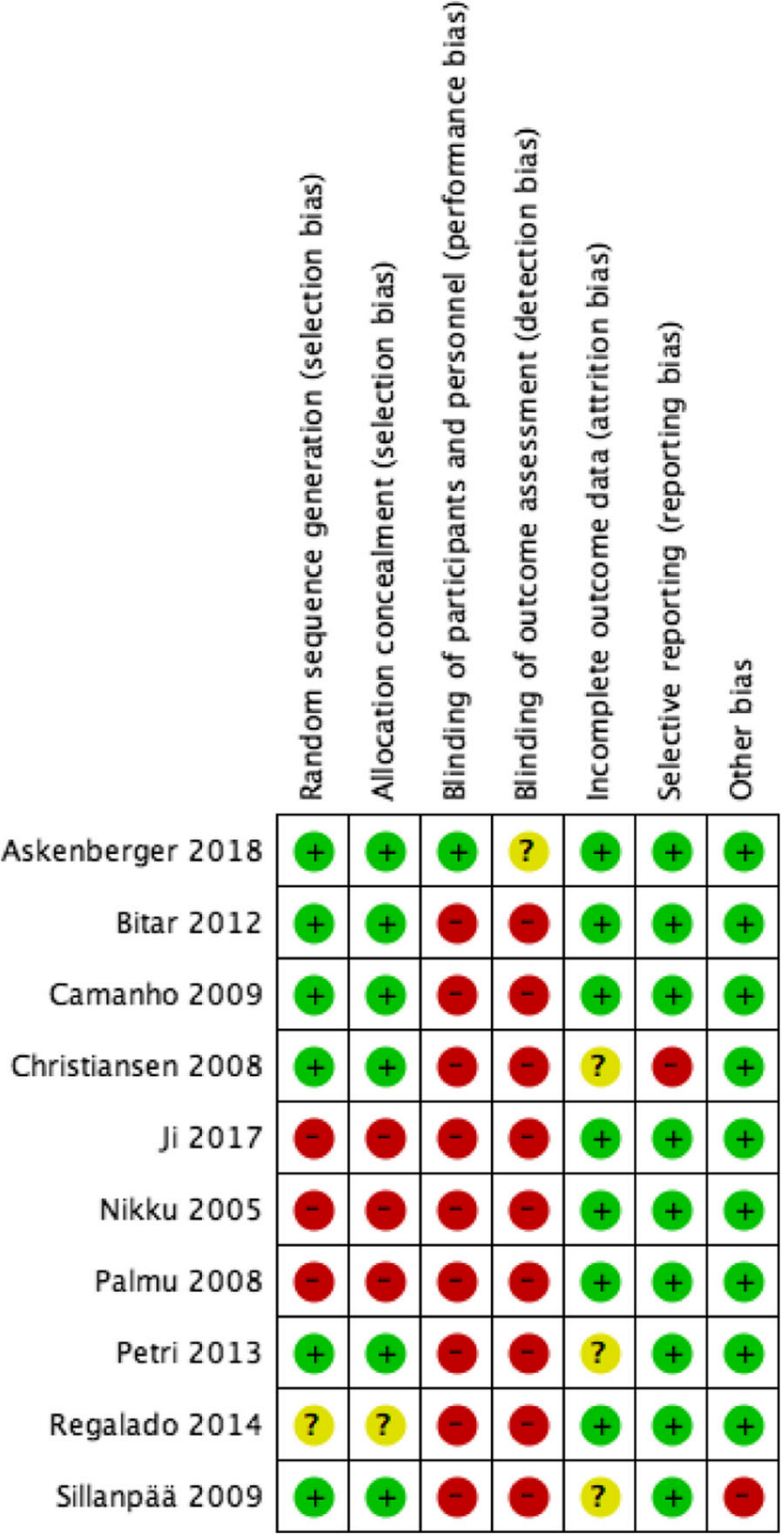
Risk of bias summary: review authors’ judgements about each risk of bias item for each included study

The search strategy is exhibited in Fig. 1. In total, the literature search identified 355 potentially relevant papers. Ten RCTs [6, 20–28] met the inclusion criteria after removing duplicates, scanning the titles and abstracts, and reviewing the full texts. Among all the participants in the 10 RCTs, 297 patients were treated with a surgical procedure, while 263 were treated with conservative therapy. The details of each RCT are shown in Table 1. In cases of operative treatment, the surgical interventions were noted. The risk of bias of each RCT is provided in Figs. 2 and 3.

**Table 1:** Characteristics of studies included in this meta-analysis

Study	Surgical interventions	Gender		Sample size		Mean age		Follow-up
		Male	Female	SG	CG	SG	CG	
Nikku et al. [20]	MR repair, MPFL augmentation, LRR	45	82	70	57	20	20	7.2 years
Christiansen et al. [21]	MPFL repair	42	35	42	35	20	19.9	2 years
Palmu et al. [22]	MPFL repair or LRR	18	46	36	28	13	13	14 years
Camanho et al. [23]	MPFL repair	13	20	17	16	24.6	26.8	40.4 m
Sillanpää et al. [24]	MR reefing or Roux-Goldthwait	37	3	18	22	20	20	7 years
Bitar et al. [25]	MPFL reconstruction	21	20	21	20	24	24	44 m
Petri et al. [6]	MR repair or LRR	13	7	12	8	27.2	21.6	2 years
Regalado et al. [26]	LRR or Modified Roux-Goldthwait	14	22	16	20	13.5	13.5	6 years
Ji et al. [27]	MPFL repair	20	36	30	26	-	-	42 m
Askenberger et al. [28]	MPFL repair	38	36	37	37	13.2	13	2 years

*SG* surgical group, *CG* conservative group, *MR* medial retinaculum, *LRR* lateral retinacular release, *MPFL* medial patellofemoral ligament

### Recurrent dislocation rate

**Fig. 4:**
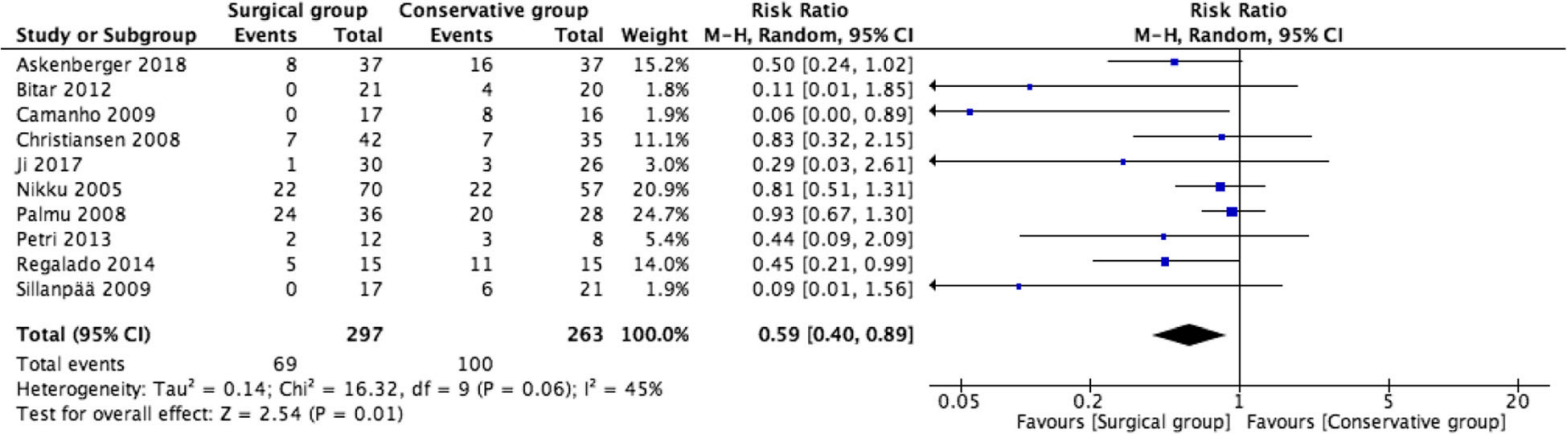
Forest plot of recurrent dislocation rate between two groups

Data on recurrent dislocation rate was available in all RCTs, and the recurrent dislocation rate was statistically higher in the conservative group according to the pooled analysis (RR = 0.59, 95% CI 0.40–0.89; *P* = 0.01, *I*^2^ = 45%) (Fig. 4).

### Subluxation rate

**Fig. 5:**
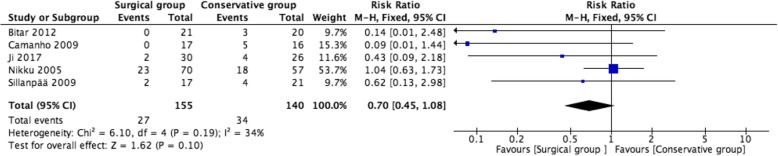
Forest plot of subluxation rate between two groups

Five studies reported the subluxation rate. The pooled analysis did not reveal any significant difference in the subluxation rate of the surgery group versus the conservative treatment group (RR = 0.70, 95% CI 0.45–1.08, *P* = 0.10), without significant heterogeneity (*P* = 0.19, *I*^2^ = 34%) (Fig. 5).

### Kujala score

**Fig. 6:**
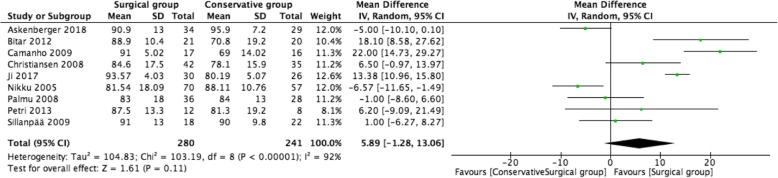
Forest plot of Kujala score between two groups

The Kujala scores were informed in nine studies involving 521 patients. There was no statistically significant difference in Kujala scores between the two groups (MD = 5.89; 95% CI − 1.28, 13.06; *P* = 0.11, *I*^2^ = 92%) (Fig. 6).

### Tegner activity score

**Fig. 7:**
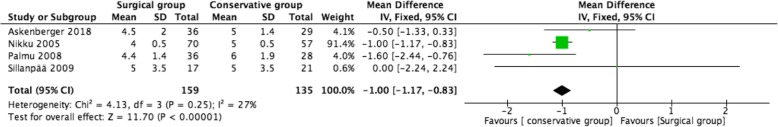
Forest plot of Tegner activity score between two groups

Four studies with a sum of 294 patients covered the Tegner activity score. Tegner activity score in the conservative treatment group was statistically higher than in the surgery group grounded on the pooled analysis (MD = − 1.00; 95% CI − 1.17, − 0.83; *P* < 0.00001), without significant heterogeneity (*P* = 0.25, *I*^2^ = 27%) (Fig. 7).

### Frequency of reoperation

**Fig. 8:**
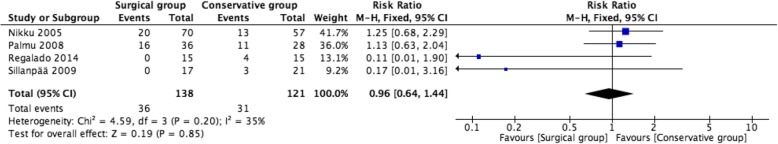
Forest plot of reoperation between two groups

Four trials involving 259 patients provided data on the frequency of reoperation. No statistically significant difference was discovered between the two groups (RR = 0.96, 95% CI 0.64–1.44, *P* = 0.85), without significant heterogeneity (*P* = 0.20, *I*^2^ = 35%) (Fig. 8).

### Patient satisfaction

**Fig. 9:**
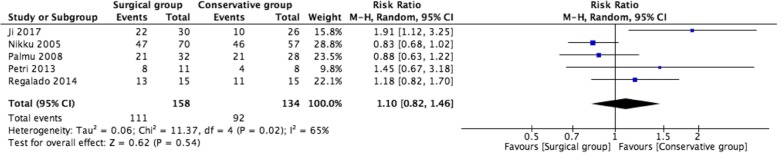
Forest plot of patient’s satisfaction between two groups

Five studies recorded patient satisfaction data. Figure 9 shows the number of patients that was satisfied with the outcome (satisfaction rating of excellent or good). The pooled data showed no significant difference between conservative treatment and surgery (RR = 1.10, 95% CI 0.82–1.46, *P* = 0.54) with moderate heterogeneity (*P* = 0.02, *I*^2^ = 65%) (Fig. 9).

## Discussion

### Advantages

The present meta-analysis was conducted in order to update the RCT-based evidence of the outcomes of conservative versus surgical treatment for APPD. The main findings of this research are that both the recurrent dislocation rate and Tegner activity score were significantly lower after surgery in comparison with conservative therapy. The meta-analysis provides evidence to suggest that conservative treatment for APPD improves patient outcomes. It can ensure the quality of the meta-analysis to follow steps of Cochrane system evaluation production and principles of PRISMA guides. It can effectively reduce the selection bias to include only randomized controlled study. This study has made a clear literature into and exclusion criteria, and the included studies were strict quality evaluation. Statistically, the application of the RevMan 5.3 software according to the Cochrane standard guarantees the quality of research.

### Efficacy evaluation

Tegner activity score was significantly higher in the conservative treatment group compared with surgery group, which is different from the outcome proposed by Smith et al. [29]. The Tegner score is a modified performance test used for monitoring rehabilitation progress and evaluating the patients’ physical activities [30]. In three of the four included studies that reported Tegner scores, the scores were reduced at follow-up, while in the remaining study the scores were unchanged. The reason may be that surgical treatment is excessive to repair the patellar soft-tissue stabilizers, which may result in overtightening tissue integrity and the compete loss of residual laxity. Residual laxity of the injured structure, defined objectively by an increase in passive lateral excursion of the patella, may contribute to a higher Tegner score seen with surgery [19]. Nikku et al. [20] and Palmu et al. [22] also noted that primary repairing the injured medical retinacular structures might not improve knee function outcome in the long run. Muscle function can be another reason [28]. The most common complication following knee surgery is reduced range of motion, with accompanying pain over the medial retinaculum or in the patellofemoral joint in up to 30% of cases [31]. Surgery can make rehabilitation insufficient, resulting in weaker muscles. Psychological factors are another reason. Children and adolescents generally are more active in sports and participate in competitive sports more frequently than adults do in Finland [22]. These patients, especially children, have never had a knee injury before and are not familiar with what an unstable knee can do to them. This may affect the motivation for surgery and rehabilitation expectations.

The pooled data analysis showed that the surgery group had a significantly lower recurrent dislocation rate than the conservative treatment group, which is consistent with Smith’s conclusion [29]. Interestingly, among all the included studies, Bitar’s study was the only one which showed a decrease in the recurrent dislocation rate after surgery, while the other studies showed no statistical difference in the recurrent dislocation rate. However, the recurrence rate of dislocation in the non-surgical group increased significantly after data aggregation. Moreover, predisposing factors for APPD were not equally distributed in the two treatment arms in most of the studies [18]. The anatomic factors that can predispose a patient to recurrent patellar instability include patella alta, genu valgum, torsional deformity, lateral patellar tilt, trochlear dysplasia, increased Q-angle, and elevated tibial tubercle–trochlear groove (TT–TG) distance [8]. Many surgeons recommend primary osseous procedures with or without MPFL reconstruction in those with high-grade trochlear dysplasia [32], while reconstruction of MPFL alone is the accepted method to restore medial stability in patients without anatomic abnormalities who frequently experience patellar dislocation [33].

No significant difference was detected in the field of Kujala score between the two groups, which is also consistent with Smith’s conclusion [29]. However, the present findings should be interpreted with caution because of the considerable heterogeneity. This may have been caused by factors such as the study design, demographic characteristics, intervention treatments, follow-up duration, and even the scoring system itself. Kujala score is the most commonly used method to evaluate patellofemoral joint disease in recent years [12]. Whereas, there is a growing debate about whether the scoring adequately reflects the complexity of patellar instability [34]. A new assessment score called the Norwich Patellar Instability score has been implemented to overcome the weakness of the Kujala score [35] and it is recommended for use in future studies [12]. In addition, Bitar et al. concluded that the Kujala score was significantly increased by MPFL reconstruction [25]. Their trial is the first to compare conservative treatment with MPFL reconstruction, rather than MPFL repair [19]. Hence, MPFL reconstruction may be the effective mainstay of surgical treatment of APPD.

### Limitations

The present meta-analysis updated the previous literature. However, the meta-analysis also has limitations. Firstly, all the included studies showed some weaknesses, mainly related to low-quality RCT design regarding factors such as age, gender, surgical, and rehabilitative interventions. Three studies [22, 26, 28] reported solely on patients younger than 16 years, one study [27] contained no information on patient age, and the remaining six studies included patients of varying ages, including children, adolescents, and adults; this may have affected the outcomes, as age is reportedly correlated with the incidence of primary and recurrent patellar dislocation [36]. Secondly, the risk of bias scores reflects that some studies are at high risk, although all data were extracted from RCTs. We attempted to resolve the problem by performing a subgroup analysis by type of surgery. However, it is difficult to compare studies using a single surgical technique with studies using multiple surgical methods to treat APPD. In the study by Nikku et al. [20], 63 patients had the medial retinaculum in a variety of ways: suture, duplication, or additional MPFL augmentation. Among them, 54 underwent a LRR. Seven patients underwent only a LRR. In the study by Palmu et al. [22], MPFL repair was performed in 29 knees, of which 25 had a LRR. Seven patients underwent LRR alone. Petri et al. [6] and Regalado et al. [26] realigned the extensor mechanism with the Roux-Goldthwait procedure, which is used to manage recurrent patellar dislocation [37]. It is unclear why APPD was often treated with more than one type of surgical technique several years ago. One possible reason for this is that this type of injury is relatively uncommon, and no surgical method has been definitively proven to be effective. Thus, various procedures were performed simultaneously in order to surgically rectify an MPFL rupture and subsequent patellar instability. Another possible reason is that patients with APPD are often linked with anatomic abnormality, and those congenital malformations may need to be corrected during ligamentous repair in order to improve outcomes. In a word, surgical confounders discussed above should also be considered in future clinical trials. Previous meta-analyses have also discussed other limitations, including the small number of trials, diverse demographics, and lack of allocation concealment methods [15–19].

## Conclusions

The present meta-analysis found that despite a very high recurrent dislocation rate, the majority of patients who received conservative treatment were content with the function of their knees; conservative treatment may produce better outcomes than surgery for APPD in consideration of Tegner activity score. However, in view of the limited number of the studies available, the findings should be interpreted with caution. More convincing evidence is required to confirm the effect of surgery, especially MPFL reconstruction.

## Data Availability

As a meta-analysis, there are no patient data sets.

## Abbreviations

RCTs: Randomized controlled trials
APPD: Acute primary patellar dislocation
MPFL: Medial patellofemoral ligament
PRISMA: Preferred Reporting Items for Systematic Reviews and Meta-Analyses
MD: Mean difference
CI: Confidence intervals
RR: Risk ratio
SG: Surgical group
CG: Conservative group
MR: Medial retinaculum
LRR: Lateral retinacular release
